# Severe Environmental Contamination and Elevated Blood Lead Levels Among Children — Zambia, 2014

**Published:** 2014-11-07

**Authors:** Jack Caravanos, Richard Fuller, Stephan Robinson

**Affiliations:** 1 City University of New York School of Public Health, New York, NY; 2 PureEarth/Blacksmith Institute, New York, NY; 3 Green Cross Switzerland, Zürich, Switzerland

Lead poisoning can have devastating health consequences, especially for children, with childhood lead exposure estimated to contribute to 600,000 new cases globally of children with intellectual disabilities every year. Lead exposure is entirely preventable, yet is estimated to account for 0.6% of the global burden of disease, with the highest burden in developing regions (1). Kabwe, the second largest city in Zambia with a population of approximately 203,000, is located in Zambia’s Copperbelt. During 1904–1994, lead mining and smelting operations contaminated the soil in residential areas, but no extensive environmental health assessment was completed (2). In 2003, the World Bank funded the Copperbelt Environmental Project to assist the Government of Zambia in addressing environmental health problems related to the mining sector. Components of the project included removal of mining waste materials, soil remediation, resident evacuation, and treatment of lead-exposed children. During July 22–28, 2014, a team from PureEarth/Blacksmith Institute, the City University of New York School of Public Health, and Green Cross Switzerland conducted extensive surface soil testing and blood lead testing of children in six communities adjacent to the now-closed Kabwe mines and smelters.

Surface soil lead concentrations were measured at 339 locations in residential areas using an X-ray fluorescence spectrometer. The approximately 4 km^2^ sampling area encompassed 12 residential neighborhoods near the abandoned smelters and mine. Surface soil lead concentrations ranged from 139 mg/kg to 62,142 mg/kg, with a geometric mean concentration of 1,470 mg/kg. The highest results in soil were found in neighborhoods directly adjacent to the abandoned smelters. Of the 339 soil tests, 86 readings (25.4%) were above the U.S. Environmental Protection Agency lead in soil guidance value of 400 mg/kg (3), and 98% were above the Zambia guideline of 200 mg/kg. In comparison, lead concentrations in 25 surface soil samples taken in the capital city of Lusaka ranged from 12 mg/kg to 66 mg/kg.

In addition to soil testing, 196 children aged 2–8 years living within these communities were tested for blood lead using a LeadCare II blood testing system (Magellan Diagnostics, Inc., N. Billerica, Massachusetts) under the supervision of the district health center. The system uses capillary blood, and children’s fingers were thoroughly cleaned before testing. The mean blood lead level (BLL) was 48.3 micrograms per deciliter (*μ*g/dL) of whole blood. The lowest BLL measured was 13.6 *μ*g/dL. The upper BLL of detection by the testing system is 65.0 *μ*g/dL; 52 (26.5%) readings exceeded that limit. The upper value for the CDC reference range for BLLs in children is 5 *μ*g/dL (2). CDC recommends that lead chelation therapy be considered when a child has a BLL ≥45 *μ*g/dL.* Previous World Bank funding provided modest case-management assistance and chelation therapy for severe cases of lead poisoning; however, such efforts have been suspended and are currently unavailable.

Reports of lead poisoning from mining, smelters, and battery processing operations in other low-income countries demonstrate the severity of lead poisoning in children (4). In a review of 242 studies of known chemically contaminated sites, lead was the primary contaminant in 57 (25%) studies, representing 8,345 exposed children (5).

The economically disadvantaged communities living near the former lead mining and smelting site in Kabwe are at significant risk from lead contamination. Additional hotspot remediation and mine tailings dust control measures should be considered as a primary preventive measure. More urgent is the implementation of a BLL surveillance and treatment program for affected children and behavioral and educational interventions to reduce the extent of the poisoning and prevent continued exposure.

## Footnotes

* Additional information available at http://www.cdc.gov/nceh/lead/acclpp/blood_lead_levels.htm.
